# Association between dialysis effluent leukocyte count after initial antibiotic treatment and outcomes of patients with peritoneal dialysis-associated peritonitis: a retrospective study

**DOI:** 10.1080/0886022X.2023.2258990

**Published:** 2023-09-22

**Authors:** Tao Hong, Xiaoxia Wang, Shangmei Li, Liping Zhai, Na Wu, Haijuan Yang, Cuiwei Yao, Huafeng Liu

**Affiliations:** Department of Nephrology, Affiliated Hospital of Guangdong Medical University, Zhanjiang, China

**Keywords:** Peritoneal dialysis-associated peritonitis, peritoneal dialysis effluent leukocyte count, mortality, treatment failure, refractory peritonitis

## Abstract

**Background:**

Among patients with peritoneal dialysis-associated peritonitis (PDAP), It has been regarded as an indicator of deterioration of clinical condition that peritoneal dialysis effluent leukocyte count (PDELC) cannot be restored to normal after initial antibiotic therapy. However, the precise relationship between PDELC on day 5 and the clinical outcomes of PDAP episodes remains uncertain.

**Aims:**

To explore the association between PDELC on day 5 and clinical outcomes of PDAP episodes.

**Methods:**

This retrospective study was based on the medical chart database of the Affiliated Hospital of Guangdong Medical University. Multivariable regressions were used to evaluate the association between PDELC on day 5 and 60-day mortality, half-year mortality, treatment failure, and the length of stay in hospital with adjustment for confounding factors.

**Results:**

A total of 549 PDAP episodes in 309 patients were enrolled. The total 60-day mortality, half-year mortality, and rate of treatment failure was 6.0%, 9.8%, and 14.2%, respectively. Compared with patients with normal PDELC, those with PDELC ≥2000 × 10^6^/L on day 5 had significantly higher 60-day mortality (31.1% vs 2.7%), half-year mortality (35.6% vs 5.6%), and treatment failure (46.7% vs 5.7%). In multivariate adjusted regression, the ORs (95%CI) were 6.99 (2.33, 20.92; *p* = 0.001), 4.97(1.93, 12.77; *p* = 0.001), and 5.77 (2.07, 16.11; *p* = 0.001), respectively. Patients with PDELC were 100–2000 × 10^6^/L on day 5 had a higher rate of treatment failure than those with normal PDELC (26.9% vs 5.7%) (OR = 3.03, 95%CI 1.42, 6.46; *p* = 0.004). After sensitivity analysis, the results remained robust.

**Conclusions:**

Among patients with PDAP, increased PDELC on day 5 was associated with a greater risk of 60-day mortality, half-year mortality, and treatment failure.

## Introduction

Peritoneal dialysis-associated peritonitis (PDAP) is a common serious complication of peritoneal dialysis (PD). It is not only the direct or major contributing cause of death in up to 8.6% −15% of patients on PD [1–3], but also contributes to 20% of PD technique failures, considerable morbidity, and health care costs [4–6].

In patients with PDAP, the change in the peritoneal dialysis effluent leukocyte count (PDELC) after initial antibiotic therapy has been considered as an indicator of disease severity. The guidelines define refractory peritonitis as failure of the PD effluent to clear after 5 days of appropriate antibiotic treatment, which is considered to deteriorate the clinical condition, and recommend PD catheter removal in this situation [7]. However, the cutoff of PDELC >100 × 10^6/^L after 5 days of antibiotic therapy may be arbitrary reference tool. In our practice, we observed that some patients with refractory PDAP, who were treated with antibiotics alone and without catheter removal, could recover from peritonitis. Furthermore, the exact relationship between PDELC on day 5 and the clinical outcomes of PDAP episodes remains unclear. Therefore, we conducted a retrospective cohort study to determine the association between PDELC on day 5 and outcomes to provide an optimal reference for clinicians.

## Methods

### Data sources and setting

This retrospective cohort study was conducted using the medical chart database of the Affiliated Hospital of Guangdong Medical University, a regional center institution in South China. We obtained approval to access this database and complied with all relevant ethical regulations regarding the use of the data for our study.

### Study population

Patients who fulfilled the diagnostic criteria for PDAP and were hospitalized at the PD center of the Affiliated Hospital of Guangdong Medical University were eligible for our study. Data from December 1, 2014, to January 31, 2023, were retrospectively collected from medical charts. In accordance with the newly updated ISPD Peritonitis Guidelines, PDAP was diagnosed when at least two of the following were present:1) clinical features consistent with peritonitis, that is, abdominal pain and/or cloudy dialysis effluent; 2) PDELC > 100 × 10^6^/L, with >50% polymorphonuclear leukocytes (PMN); and 3) positive dialysis effluent culture [7]. All PDAP episodes, including primary, relapse, repeat, and recurrent infections, were collected. The exclusion criteria were as follows:1) patients younger than 10 years of age; 2) absence of data on PD fluid culture; 3) absence of data on PDELC on day 5; 4) absence of data on clinical outcomes; 5) those patients who had been treated with antibiotics before hospitalization; 6) episodes of peritonitis that occurred after PD catheter removal; and 7) comorbidities of malignant tumor or gastrointestinal perforation.

The protocol of antibiotic treatment for PDAP was as follows:1) Empirical antibiotic therapy was initiated as soon as possible, using the intraperitoneal route, after appropriate microbiological specimens had been obtained. 2) Empirical antibiotic regimens covered both Gram-positive and Gram-negative bacteria. Gram-positive organisms were covered by a first-generation cephalosporin or vancomycin, and gram-negative organisms by a third-generation cephalosporin or aminoglycoside. The antimicrobial regimen was adjusted according to the drug sensitivity results, and antibiotic treatment duration was determined according to the ISPD guidelines [7]. 3) In cases of refractory PDAP, the strategy of prolonged (or adjusted) antibiotic therapy or catheter removal was decided by clinicians depending on the patient’s clinical conditions.

### Study variables

We extracted from the medical chart the demographic data, medical history, laboratory test data within the first 2 days after admission, and organisms culture data, including age, sex, PD duration, modality of PD (APD or CAPD), etiology of end-stage renal disease, comorbidities, hemoglobin (HB), white blood cell count (WBC), albumin, serum potassium, serum phosphorus, C-reactive protein (CRP), procalcitonin (PCT), PDELC on day 1, PDELC on day 5, and result of effluent organism-culture. The median value was obtained from multiple measurements of biochemical indices.

### Outcomes

The primary independent variable was PDELC on day 5, defined as peritoneal dialysis effluent leukocyte count after 5 days of antibiotic treatment. The primary outcome of this study was 60-day mortality, which was defined as death occurring within 60 days from the date of PDAP occurrence. Secondary outcomes included the half-year mortality, treatment failure, and the length of stay (LOS) in hospital. Half-year mortality was defined as death within half years from the date of PDAP occurrence. Treatment failure included PD catheter removal, permanent switch to hemodialysis, or PDAP-related death.

### Statistical analysis

A descriptive analysis was performed for all participants. Categorical variables are expressed as numbers and percentages. Continuous variables are expressed as mean and standard deviation (SD) for normal distributions or median and interquartile range for skewed distributions. We used the chi-square test, T-test, and Kruskal-Wallis test to compare categorical, normally distributed, and non-normally distributed continuous variables, respectively.

The dose-response association between the PDELC on day 5 and outcomes was implemented to determine the significant inflection point. PDAP episodes were divided into low and high PDELC on day 5 groups based on the inflection point.

Multivariable logistic regression models were applied to evaluate the relationships between PDELC on day 5 and 60-day mortality, half-year mortality, and treatment failure. The PDELC on day 5 was analyzed as a continuous and categorical variable with 100 × 10^6^/L and 2000 × 10^6^/L as the cutoff. Multivariable linear regression models were used to assess the association between PDELC on day 5 and in-hospital LOS. We used unadjusted and multivariate-adjusted models. By reviewing previous literation, the clinical meaningful variables or important risk factors reported before were considered as candidates for confounders. Then, multicollinearity in regression models was evaluated by calculating the variance inflating factor. Values of the variance inflating factor exceeding 10 were considered to indicate multicollinearity and excluded. Then, to assess confounding, we entered covariates into a regression model in the basic model or eliminated the covariates in the complete model one by one and compared the regression coefficients. Those covariates altering initial regression coefficients by more than 10% were included. Finally, the clinical meaningful variables or ones that affected the regression coefficients by more than 10% were selected as confounders to adjust in the final model. In this study, the regression models were adjusted for age [8–10], PD duration [10–13], comorbidity of diabetes mellitus [8,13–16], serum albumin level [10,16], CRP level [17], fungal peritonitis [6,13,18], Gram-negative bacteria peritonitis [6,19], and multi-organism peritonitis [6,18–20].

By drawing the receiver operating characteristic (ROC) curve, the predictive value of PDELC for the clinical outcomes was evaluated.

The baseline characteristics of included cases and excluded cases, which were excluded due to missing the data of PDELC on day 5, were compared and displayed in the supplementary materials (Table S1). Among included cases, the details of missing data were displayed in the supplementary materials (Table S2). We used multiple imputation, based on three replications and a chained equation approach method in the R mice procedure, to maximize statistical power and minimize bias that might occur account for missing data. The distributions of all the variables of missing data were of similar values to the imputation data (Table S2). We also performed sensitivity analyses using a complete-case analysis (We repeated all regression analyses with the complete data cohort for comparison) [21] (Table S1).

All of the analyses were performed using the statistical software packages R 3.6.0 (http://www.R-project.org, The R Foundation) and Free Statistics software version 1.4. A two-tailed test was performed, and statistical significance was set at *p* < 0.05.

### Sensitivity analysis

The robustness of these findings was assessed in multiple sensitivity analyses. First, subgroup analyses were conducted according to age, PD duration, serum albumin level, CRP level, PDELC on day 1, fungal peritonitis, and Gram-negative bacteria peritonitis with adjusted confounders. Second, we restricted the cohort to the episodes with culture-positive results by excluding episodes with culture-negative and repeated the regression analyses. Moreover, we repeated the regression analysis, excluding episodes with MRSA, Pseudomonas spp., and fungal peritonitis because they have high treatment failure or are associated with poor outcomes. In addition, unmeasured confounding may still exist. A sensitivity analysis can be performed to evaluate the strength required of a potential unmeasured confounder to change the association. VanderWeele and Ding [22] proposed a value to measure the ‘evidence of causality’ called the E-value. We calculated E-values for our primary and secondary outcomes to assess the potential of unmeasured confounders to influence our results.

We compared the clinical characteristics of early-onset cases (defined as the occurrence of the episode within 12 months of the initiation of PD) with later-onset cases (defined as the occurrence of the episode after 12 months). The detailed information was displayed in the supplementary materials (Table S5).

## Results

### Demographic data and baseline characteristics

**Figure 1.**
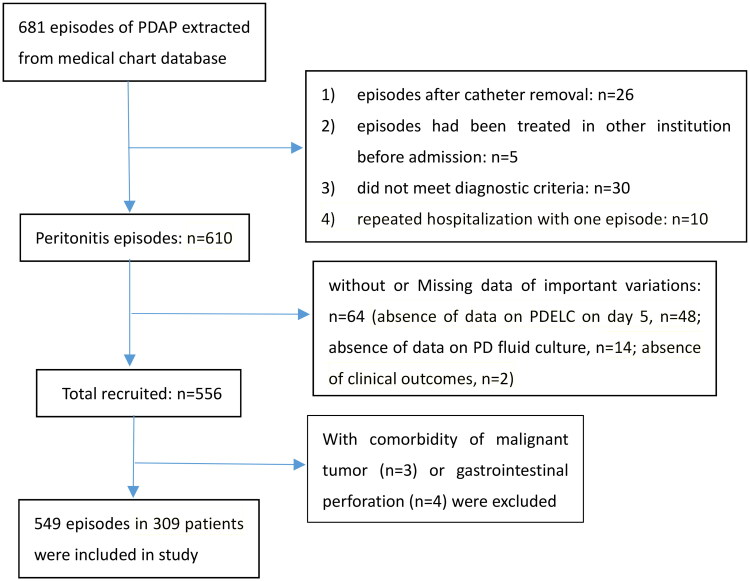
Flow chart of the study participants.

**Figure 2.**
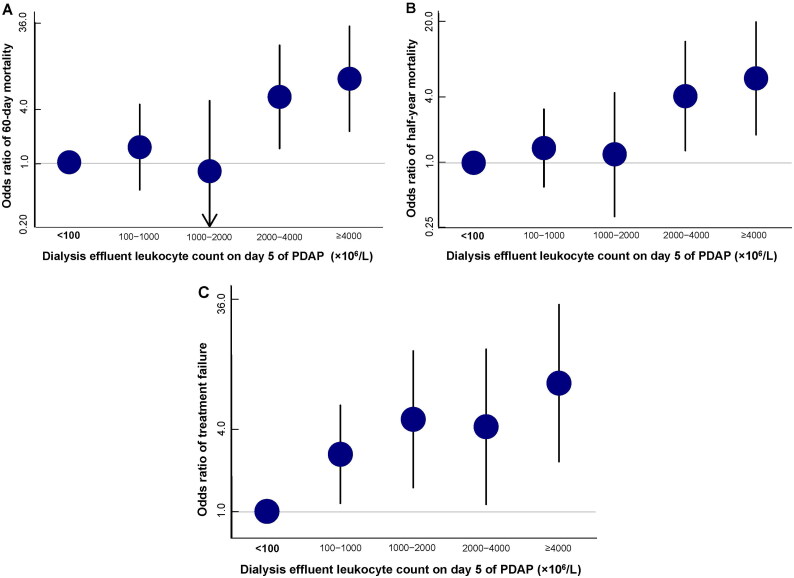
Dose-response relationship between PDELC on day 5 and outcomes, including 60-day mortality (A), half-year mortality (B), and treatment failure (C).

A total of 681 PDAP episodes were extracted from the medical chart database between December 1, 2014, and January 31, 2023. Ultimately, 549 episodes in 309 patients were enrolled in the analysis cohort. The flow diagram of the study episodes is shown in Figure 1. Dose-response relationships of PDELC on day 5 with 60-day mortality, half-year mortality, and treatment failure were found in the logistic regression model (Figure 2). Odds ratios were graphically represented and a meaningful inflection point, which was around 2000 × 10^6^/L, existed when the risk of both 60-day and half-year mortalities began to rise significantly. As for the relationship between PDELC on day 5 and treatment failure, the inflection point of PDELC-5D was approximately 100 × 10^6^/L when the risk of treatment failure began to increase significantly (Figure 2). All episodes were grouped using inflection points as cutoff values in further multivariate regression analyses.

In our PD center, most patients performed PD as the first modality for renal replacement therapy, another small part of patients accepted transient hemodialysis treatment for no more than three weeks before initiation of PD treatment. Only a small proportion of PD patients were performing automated peritoneal dialysis (APD) (about 5%) when PDAP occurred. Because most patients lived far from the PD center, once peritonitis was suspected, the patient would be admitted for inpatient treatment as soon as possible. The usual empirical initial antibiotic regimen in our center was intraperitoneal cefazolin combined with Ceftazidime. The antimicrobial regimen was adjusted according to the drug sensitivity results and the clinical effects of drugs. All patients in our center were not treated with antibiotic exit-site prophylaxis.

The baseline characteristics of all and grouped PDAP episodes are summarized in Table 1. The age of all participants was 53.4 ± 14.0, and 57.8% (317) were male. There were no significant differences in the etiology distribution between the groups of patients. APD accounted for only a small proportion in either total or grouped episodes (4.4%-5.2%). Patients in the high PDELC group (PDELC on day 5 ≥ 2000 × 10^6^/L) had a longer PD duration than those in the normal group (PDELC on day 5 < 100 × 10^6^/L) (47.9 ± 34.9 *vs* 32.0 ± 29.7 months). Furthermore, patients in the high PDELC group had more severe inflammation (higher C-reactive protein, Procalcitonin, and PDELC levels on day 1). However, there were no obvious differences in other laboratory variables (such as Hemoglobin, WBC, Albumin, Serum Potassium, as well as Serum phosphorus) between the groups. Compared with patients with normal PDELC on day 5, Patients in the high PDELC group had a lower rate of Gram-positive bacteria (20% *vs* 50.5%), and a higher rate of Gram-negative bacteria (64.4% *vs* 24.9%), fungi (24.4% *vs* 1.4%), Pseudomonas (13.3% *vs* 2.4%), and multiple organisms (15.6% *vs* 2.4%). There were four cases of MRSA and only one case of nontuberculosis mycobacterium in the cohort. The trends were clear that the 60-day mortality, half-year mortality, rate of treatment failure and catheter removal increased with an increase in PDELC on day 5 (Table 1).

**Table 1.** Baseline characteristics of 549 peritonitis episodes.

Variables	All (*N* = 549)	PDELC on day 5 (× 10^6^ cell/L)		
		<100 (*N* = 370)	100–2000 (*N* = 134)	>2000 (*N* = 45)
Sex, n (%)				
Female	231 (42.2)	161 (43.5)	49 (36.8)	21 (46.7)
Male	317 (57.8)	209 (56.5)	84 (63.2)	24 (53.3)
Age (years)	53.4 ± 14.0	53.6 ± 14.3	52.5 ± 13.9	55.2 ± 11.9
Etiology of ESRD, n (%)				
Chronic glomerulonephritis	395 (71.9)	266 (71.9)	100 (74.6)	29 (64.4)
Diabetes nephropathy	19 (3.5)	11 (3)	4 (3)	4 (8.9)
Hypertensive nephropathy	51 (9.3)	36 (9.7)	11 (8.2)	4 (8.9)
Obstructive nephropathy	48 (8.7)	30 (8.1)	14 (10.4)	4 (8.9)
Lupus nephritis	13 (2.4)	9 (2.4)	2 (1.5)	2 (4.4)
Gouty nephropathy	13 (2.4)	11 (3)	2 (1.5)	0 (0)
Chronic interstitial nephritis	5 (0.9)	3 (0.8)	0 (0)	2 (4.4)
Polycystic kidney disease	5 (0.9)	4 (1.1)	1 (0.7)	0 (0)
Comorbid conditions, n (%)				
Clinical atherosclerotic vascular disease	118 (21.5)	75 (20.3)	33 (24.6)	10 (22.2)
Diabetes mellitus	33 (6.0)	18 (4.9)	10 (7.5)	5 (11.1)
Comorbidity Infections, n (%)				
Upper respiratory tract infection	9 (1.6)	6 (1.6)	3 (2.2)	0 (0)
Pulmonary infection	110 (20.0)	60 (16.2)	33 (24.6)	17 (37.8)
Gastroenteritis	11 (2.0)	5 (1.4)	4 (3)	2 (4.4)
cholecystitis	6 (1.1)	3 (0.8)	2 (1.5)	1 (2.2)
Tunnel infection	4 (0.7)	1 (0.3)	3 (2.2)	0 (0)
sepsis	5 (0.9)	3 (0.8)	1 (0.7)	1 (2.2)
others	6 (1.1)	4 (1.1)	1 (0.7)	1 (2.2)
PD duration (months), Mean ± SD	35.3 ± 31.1	32.0 ± 29.7	40.3 ± 32.0	47.9 ± 34.9
Early-onset (<12 months), n (%)	156 (28.4)	117 (31.6)	31 (23.1)	8 (17.2)
APD, n (%)	27 (4.9)	18 (4.9)	7 (5.2)	2 (4.4)
CAPD, n (%)	522 (95.1)	352 (95.1)	127 (94.8)	43 (95.6)
Laboratory variables				
HB (g/L), Mean ± SD	103.2 ± 21.4	103.6 ± 20.6	101.4 ± 23.4	104.7 ± 21.1
WBC (×10^9^/L), Mean ± SD	10.0 ± 4.9	10.3 ± 4.8	9.5 ± 4.8	8.7 ± 5.4
Albumin (g/L), Mean ± SD	29.0 ± 5.3	29.5 ± 5.2	28.0 ± 5.5	28.2 ± 5.5
Serum Potassium (mmol/L), Mean ± SD	3.5 ± 0.7	3.5 ± 0.7	3.5 ± 0.7	3.6 ± 0.8
Serum phosphorus (mmol/L), Mean ± SD	1.5 ± 0.5	1.5 ± 0.5	1.6 ± 0.5	1.6 ± 0.5
CRP, (mg/L), Median (IQR)	88.3 (38.7, 136.5)	78.3 (31.5, 115.0)	114.0 (64.7, 180.8)	147.0 (84.2, 193.8)
PCT, (ng/ml), Median (IQR)	4.9 (1.1, 25.2)	3.8 (0.9, 19.1)	5.3 (1.3, 30.0)	25.1 (6.0, 66.9)
PDELC on day 1 (×10^6^/L), Median (IQR)	1800.0 (590.0, 4100.0)	1575.0 (507.5, 3500.0)	2055.0 (625.0, 4378.0)	4100.0 (2100.0, 6200.0)
Organisms				
Culture-negative, n (%)	132 (24.0)	93 (25.1)	36 (26.9)	3 (6.7)
Gram-positive, n (%)	240 (43.7)	187 (50.5)	44 (32.8)	9 (20)
Coagulase-negative staphylococcus	83 (15.1)	70 (18.9)	10 (7.5)	3 (6.7)
Staphylococcus aureus	18 (3.3)	6 (1.6)	9 (6.7)	3 (6.7)
MRSA	4 (0.7)	1 (0.3)	2 (1.5)	1 (2.2)
Eenterococcus	14 (2.6)	8 (2.2)	5 (3.7)	1 (2.2)
Gram-negative, n (%)	166 (30.2)	92 (24.9)	45 (33.6)	29 (64.4)
Escherichia coli	90 (16.4)	52 (14.1)	24 (17.9)	14 (31.1)
Pseudomonas	25 (4.6)	9 (2.4)	10 (7.5)	6 (13.3)
Klebsiella	29 (5.3)	17 (4.6)	8 (6.0)	4 (8.9)
Fungus, n (%)	33 (6.0)	5 (1.4)	17 (12.7)	11 (24.4)
Multi-organisms, n (%)	27 (4.9)	9 (2.4)	11 (8.2)	7 (15.6)
Clinical outcomes				
Catheter removal, n (%)	55 (10.0)	10 (2.7)	30 (22.4)	15 (33.3)
treatment failure, n (%)	78 (14.2)	21 (5.7)	36 (26.9)	21 (46.7)
60-day mortality, n (%)	33 (6.0)	10 (2.7)	9 (6.7)	14 (31.1)
Half-year mortality, n (%)	52 (9.8)	20 (5.6)	16 (12.4)	16 (35.6)
in hospital LOS (day), Median (IQR)	10.0(7.0, 15.0)	8.0(6.0, 12.0)	14.0(10.0, 20.0)	22.0(14.0, 30.0)

Abbreviations: PD, peritoneal dialysis; PDELC, peritoneal dialysis effluent leukocyte count; HB, Hemoglobin; WBC, White blood cell count; CRP, C-reactive protein; PCT, Procalcitonin; LOS: Length of stay; APD, automated peritoneal dialysis; CAPD, continuous ambulatory peritoneal dialysis.

### The association between PDELC on day 5 and mortality

The total 60-day and half-year mortality rates were 6.0% and 9.8% respectively, with 2.7% and 5.6% in the PDELC on day 5 < 100 × 10^6^/L group, with 6.7% and 12.4% in the 100–2000 × 10^6^/L group, and with 31.1% and 35.6% in the ≥2000 × 10^6^/L group, respectively (Table 1).

In the multivariate logistic regression analyses, the ORs and corresponding 95% CIs for the risk of 60-day and half-year mortality according to PDELC on day 5 as continuous variable, PDELC on day 5 < 100 × 10^6^/L between 100–2000 × 10^6^/L, and ≥2000 × 10^6^/L are summarized in Table 2. Every 100 × 10^6^/L of risen PDELC increased the risk of both 60-day and half-year mortality by 3% (OR 1.03, 95%CI 1.01–1.05, *p* = 0.001; and OR 1.03, 95% CI 1.01–1.05, *p* = 0.002) in the full variable adjusted model (Model 3).

**Table 2.** Multivariable logistic regression models evaluating the association between PDELC on day 5 and clinical outcomes.

Variable	n. total	n. event (%)	Non-adjusted Model		Model 1		Model 2		Model 3	
			OR (95%CI)	P-value	OR (95%CI)	P-value	OR (95%CI)	P-value	OR (95%CI)	P-value
60-day mortality										
PDELC on day 5 (continuous), per 100 × 10^6^/L	549	33(6)	1.05(1.03,1.07)	<0.001	1.04(1.03, 1.06)	<0.001	1.03(1.01,1.05)	0.001	1.03 (1.01–1.05)	0.001
<100 × 10^6^/L	370	10 (2.7)	Ref		Ref		Ref		Ref	
100–2000 × 10^6^/L	134	9 (6.7)	2.59 (1.03, 6.53)	0.043	2.41 (0.95, 6.13)	0.064	1.32 (0.47, 3.76)	0.60	1.31 (0.46, 3.74)	0.613
≥2000 × 10^6^/L	45	14 (31.1)	16.26 (6.67, 39.61)	<0.001	14.05 (5.68, 34.74)	<0.001	6.7 (2.37, 18.90)	<0.001	6.99 (2.33, 20.92)	0.001
Half-year mortality										
PDELC on day 5 (continuous), per 100 × 10^6^/L	549	52(9.8)	1.04 (1.03, 1.06)	<0.001	1.04 (1.02, 1.06)	<0.001	1.03 (1.01, 1.05)	<0.001	1.03 (1.01, 1.05)	0.002
<100 × 10^6^/L	370	20 (5.4)	Ref		Ref		Ref		Ref	
100–2000 × 10^6^/L	134	16 (11.9_	2.37 (1.19, 4.73)	0.014	2.26 (1.13, 4.54)	0.022	1.39 (0.64, 3.03)	0.401	1.32 (0.60, 2.89)	0.486
≥2000 × 10^6^/L	45	16 (35.6)	9.66 (4.52, 20.62)	<0.001	8.58 (3.97, 18.56)	<0.001	4.5 (1.88, 10.77)	0.001	4.97(1.93, 12.77)	0.001
Treatment failure										
PDELC on day 5 (continuous), per 100 × 10^6^/L	549	78 (14.2)	1.05 (1.03, 1.07)	<0.001	1.05 (1.03, 1.07)	<0.001	1.03 (1.01, 1.05)	0.001	1.03 (1.01, 1.05)	0.007
<100 × 10^6^/L	370	21 (5.7)	Ref		Ref		Ref		Ref	
100–2000 × 10^6^/L	134	36 (26.9)	6.1 (3.41, 10.94)	<0.001	5.92 (3.28, 10.67)	< 0.001	3.27 (1.55, 6.89)	0.002	3.03 (1.42, 6.46)	0.004
≥2000 × 10^6^/L	45	21 (46.7)	14.54 (6.99, 30.26)	<0.001	13.05(6.21, 27.43)	<0.001	6.18 (2.40, 15.94)	<0.001	5.77 (2.07, 16.11)	0.001

NOTES: Adjust I model adjusts for age and PD-duration; Adjust II model adjusts for adjusts I + Fungal peritonitis + diabetes mellitus + CRP; Adjust III model adjusts for adjusts II + albumin + Gram-negative infection + multi-organism infection.

Compared with patients whose PDELC on day 5 was <100 × 10^6^/L, patients with PDELC on day 5 ≥ 2000 × 10^6^/L had a 1526% increased risk of 60-day mortality (OR = 16.26, 95%CI 6.67–39.60; *p* < 0.001) in a non-adjusted model, and had a 599% increased risk (OR= 6.99, 95%CI 2.33–20.92; *p* = 0.001) after adjusting for all covariates.

The risk of half-year mortality also increased significantly in the group of PDELC on day 5 ≥ 2000 × 10^6^/L, with a non-adjusted OR of 9.66 (95%CI 4.52–20.62; *p* < 0.001), compared with the group of PDELC <100 × 10^6^/L. After adjusting for all covariates, the OR was 4.97 (95%CI 1.93–12.77; *p* = 0.001). The statistical results were robust among all the models (Table 2).

However, compared with the group of PDELC on day 5 < 100 × 10^6^/L, there was no significant increase in 60-day (OR = 1.31, 95% CI 0.46–3.74; *p* = 0.613) and half-year mortality (OR = 1.32, 95% CI 0.6–2.89; *p* = 0.486) in the group of 100–2000 × 10^6^/L after adjusting for all the covariates.

### The association between PDELC on day 5 and treatment failure

The total treatment failure rate was 14.2%, with 5.7% in the PDELC on day 5 < 100 × 10^6^/L group, with 26.9% in the 100–2000 × 10^6^/L group, and with 46.7% in the ≥2000 × 10^6^/L group (Table 1).

In the multivariate Logistic regression analysis, compared with those in the group of PDELC on day 5 < 100 × 10^6^/L, patients in the group of 100–2000 × 10^6^/L had a significant increased risk of treatment failure, with a non-adjusted OR of 6.1 (95%CI 3.41–10.94; *p* < 0.001) and a fully adjusted OR of 3.03 (95%CI 1.42–6.46; *p* = 0.004) (Table 2). While patients in the group of ≥ 2000 × 10^6^/L had a more remarkably increased risk of treatment failure, with a non-adjusted OR of 14.54 (95% CI 6.99–30.26; *p* < 0.001) and a fully adjusted OR of 5.77 (95% CI 2.07, 16.11; *p* = 0.001).

### The association between PDELC on day 5 and length of stay (LOS) in hospital

The median in-hospital LOS was 10.0 days (IQR:7.0–15.0), with a significant increase across the <100, the 100–2000, and the ≥ 2000(×10^6^/L) groups (*p* < 0.001) (Table 1).

The generalized multivariate linear regression analysis suggested that, after adjusted for all covariates, every risen 100 × 10^6^/L of PDELC on day 5 increased in-hospital LOS by 0.20 days (95%CI 0.15–0.25; *p* < 0.001) (Table 3). The patients in the group of 100–2000 × 10^6^/L had 3.99 days (95%CI 2.51–5.47; *p* < 0.001) longer in-hospital LOS compared with those in the group of <100 × 10^6^/L. When PDELC increased to ≥2000 × 10^6^/L, it increased to 9.28 days (95%CI 6.88–11.68; *p* < 0.001) (Table 3).

**Table 3.** Multivariable linear regression models evaluating the association between PDELC on day 5 and in hospital LOS.

Variable	n. total	Non-adjusted Model		Model 1		Model 2		Model 3	
		β(95%CI)	P-value	β (95%CI)	P-value	β(95%CI)	P-value	β(95%CI)	P-value
PDELC on day 5 (continuous), per 100 × 10^6^/L	549	0.30 (0.25,0.35)	<0.001	0.29 (0.24,0.34)	<0.001	0.20 (0.16, 0.25)	<0.001	0.20 (0.15, 0.25)	<0.001
<100 × 10^6^/L	370	Ref		Ref		Ref		Ref	
100 - 2000 × 10^6^/L	134	6.11 (4.58, 7.64)	<0.001	6.11 (4.58, 7.64)	< 0.001	4.19 (2.70, 5.68)	< 0.001	3.99 (2.51, 5.47)	< 0.001
≥2000 × 10^6^/L	45	13.42 (11.02, 15.82)	<0.001	13.16 (10.75, 15.56)	< 0.001	9.47 (7.10, 11.84)	< 0.001	9.28 (6.88, 11.68)	< 0.001

NOTES: Adjust I model adjusts for age and PD-duration; Adjust II model adjusts for adjusts I + Fungal peritonitis + diabetes mellitus + CRP; Adjust III model adjusts for adjusts II + albumin + Gram-negative infection + multi-organism infection.

Because the LOS in hospital was not a normally distributed variable, we repeated the multivariate linear regression analysis using Logarithmic transformed in hospital LOS (ln(LOS)), which was a normally distributed variable. The result of multivariate linear regression analysis using ln(LOS) remained robust and was displayed in supplementary materials (Table S6).

### Sensitivity analysis

We performed sensitivity analyses using a complete-case analysis (We repeated all regression analyses with the complete data cohort for comparison). The results were robust and displayed in supplementary materials (Tables S3, S4).

**Figure 3.**
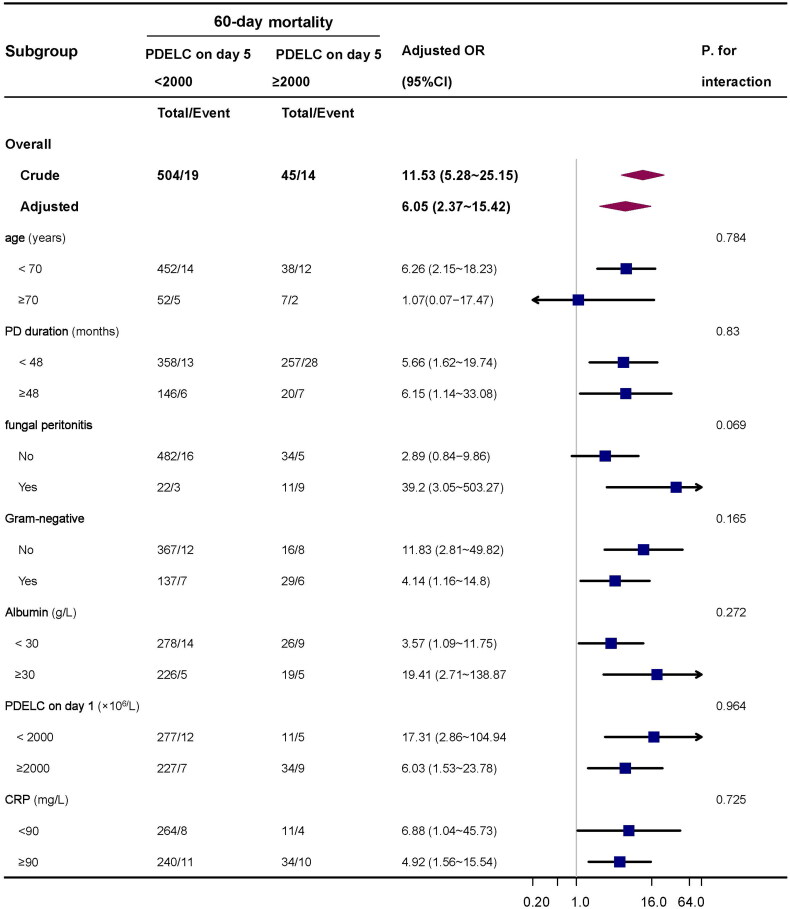
Subgroup analyses of the association between PDELC on day 5 (×10^6^/L) and 60-day mortality.

Stratified analyses were performed to examine whether the association between PDELC on day 5 and 60-day mortality was stable among the different subgroups. None of the variables, including age(<70 years), PD duration (<48 months and ≥48 months), ALB (<30 g/L and ≥30 g/L), CRP (<90mg/L and ≥90mg/L), fungal peritonitis (Yes and No), and PEDLC on day 1 (< 2000 × 10^6^/L and ≥2000 × 10^6^/L), and Gram-negative bacteria peritonitis (Yes and No) significantly affected the association between PDELC and 60-day mortality (Figure 3). Subgroup analysis showed that the relationship remained robust and reliable.We restricted the cohort to the episodes with culture-positive result by excluding those with culture-negative result and repeated all logistic regression analyses. Moreover, we also repeated logistic regression analysis after excluding episodes with MRSA, Pseudomonas spp., and fungal peritonitis. The results of regression analysis were robust and displayed in supplement (Tables S7, S8).

We calculated E-values for our primary and secondary outcomes to assess the potential of unmeasured confounders to influence our results. The primary findings were robust, unless an unmeasured confounder existed with an OR higher than 13.46 (Tables S9, S10 in Supplement).

We evaluate the predictive value of PDELC on day 5 for the clinical outcomes by drawing the receiver operating characteristic (ROC) curve. The resulting area under the ROC curve was 0.771 (95% CI 0.674–0.868) for PDELC predicting 60-day mortality, was 0.730 (95% CI 0.654–0.807) for predicting half-year mortality, and was 0.802 (95% CI 0.748–0.856) in predicting treatment failure. The figures of ROC-curve with threshold information were displayed in the supplement (Figure S1).

## Discussion

In this retrospective study, we investigated the relationship between PDELC on day 5 and clinical outcomes in patients with PDAP, taking into account competing clinical risk factors. Overall, an PDELC on day 5 increased beyond 2000 × 10^6^/L was associated with a significantly higher risk of 60-day and half-year mortality. An PDELC on day 5 increased beyond 100 × 10^6^/L was linked to an obviously greater risk of treatment failure. An increase in PDELC on day 5 was also associated with a longer LOS in the hospital. These results remained robust after adjusting for confounding variables and subgroup analyses.

Recently, an increasing number of studies have recognized that PDELC after initial antibiotic treatment may be an indicator of outcomes in patients with PDAP. Chow et al. reported that a peritoneal dialysate leukocyte count ≥1090 × 10^6^/L on day 3 was a predictor of treatment failure [23]. Xu et al. reported that a transient early increase in the effluent leukocyte count is possibly a sign of delayed response to antibiotics, but not worse outcomes [24]. Nochaiwong et al. [15] and Liu et al. [12] reported that dialysate leukocyte count >100 × 10^6^/L on day 5 is a predictor of treatment failure and catheter removal in patients with PDAP. In the ISPD guideline 2022s, refractory peritonitis was defined as failure of the PD effluent to clear after 5 days of appropriate antibiotics, and PD catheter removal was recommended for this situation to prevent morbidity and mortality associated with treatment failure, although the evidence underpinning this recommendation is limited [7]. In our center, We had noticed that, some of patients with refractory PDAP could recover from peritonitis although without being removed catheter, which was also be reported by other studies [18]. There are some reasons for clinicians choose to delay the catheter removal, such as patients’ wish, the inefficient of doctor-patient communication, difficulty of re-placing the PD catheter, and the practical experience of clinicians. In the practice of some PD center, clinicians chose to prolong antibiotic therapy and decided the timing of catheter removal depending on the clinical conditions, such as the trends of change in PDELC, present of septic shock, and fungal culture results, while a cutoff value of PDELC on day 5 > 100 × 10^6^/L in deciding catheter removal may be an arbitrary reference tool. Thus, it is highly debatable whether the catheter removal on day 5 is an optimal strategy in patients with refractory PDAP. Our study tried to explore the precise relationship between PDELC on day 5 and poor clinical outcomes. The exact PDELC value was used to define a threshold for an significantly increased risk of death or treatment failure. The results show that increased PDELC on day 5 can powerfully predict the significant rise of mortality and the rate of treatment failure, which suggests that clinicians should be more cautious when choosing to delay the catheter removal especially when the PDELC on day 5 exceeding 2000 × 10^6^/L.

Many previous studies have reported the risk factors for the poor clinical outcomes in patients with PDAP. Through reviewing previous literatures and assessing the confounding, the final regression models were adjusted for age [8–10], PD duration [10–13], comorbidity of diabetes mellitus [8, 13–16], serum albumin level [10,16], CRP level [17], fungal peritonitis [6,13,18], Gram-negative bacteria peritonitis [6,19], and multi-organism peritonitis [6,18–20]. However, the potential for residual confounding may still exist. We calculated E-values for our primary and secondary outcomes to assess the potential of unmeasured confounders to influence our results. In our study, PDELC on day 5 ≥ 2000 × 10^6^/L was associated with an increase in 60-day mortality by multivariable analysis in the full adjusted model (OR= 6.99 [95% CI 2.33–20.92]). The E-value was OR >13.46, meaning that residual confounding could explain the observed association if there exists an unmeasured covariate having an OR ≥ 13.46 with both 60-day mortality and PDELC on day 5 (see the Table S9 in supplementary materials). Significant known and measured risk factors for 60-day mortality within the multivariable logistic regression model included age (OR= 1.01 [95% CI, 0.98–1.05]), PD duration (OR= 1.01 [95% CI, 1–1.02]), diabetes mellitus (OR= 1.48 [95% CI, 0.38–5.71]), serum albumin (OR= 0.98 [95% CI, 0.91–1.05]), CRP (OR= 1.0 [95% CI, 1–1.01]), fungal peritonitis (OR= 5.98 [95% CI, 1.86–19.23]), multi-organisms peritonitis (OR= 1.47 [95% CI, 0.38–5.72]), Gram-negative organisms (OR= 0.87 [95% CI, 0.33–2.28)(See the Table S10 in supplementary materials). Therefore, it is unlikely that an unmeasured or unknown confounder would have a substantially greater effect on 60-day mortality (OR exceeding 13.46) than these known risk factors.

In the subgroup analysis, the results were robust in both the longer and shorter PD duration groups, fungal and non-fungal peritonitis groups, CRP <90mg/L and ≥90 mg/L groups, and normal and lower serum albumin level groups, Gram-negative and non-Gram-negative bacterial groups. However, in the fungal subgroup, the effect of high PDELC on mortality was more powerful than that in the non-fungal subgroup, suggesting that fungal peritonitis was also an important variable linked to the outcome. In addition, we found that high PDELC had a weaker effect on mortality in patients aged ≥70 years than those aged <70 years, which might be due to more non-peritonitis-associated deaths in elderly patients.

The reason for the high PDELC on day 5 being associated with higher 60-day and half-year mortality in patients with PDAP remains unclear. Generally, the peak of PDELC is supposed to occur on day 1 of the peritonitis because host and peritoneal immune responses are activated within several hours of bacterial invasion, with cytokine levels subsequently declining together with bacterial elimination [24–26]. A higher PDELC on day 5 indicates a poor response to antibiotic therapy and more severe inflammatory reaction [18,24]. In this study, we also observed a positive correlation between PDELC on day 5 and biomarkers of inflammation severity, such as C-reactive protein and procalcitonin (Table 1); However, in our PD center, it was observed that there were a few patients (about 2.5%), whose PDELC was transiently normal (<100 × 10^6^/L) on day 5 but later increased again, with clinical outcomes worse than those with PDELC maintained normal. This was consistent with a report by Rong Xu [9].

Our study had several important limitations. First, the data from the medical chart database of the Affiliated Hospital of Guangdong Medical University, which is a single-center retrospective cohort database, might lead to selection biases of treatment and patients. Nonetheless, we applied rigorous inclusion and exclusion criteria, and compared the baseline characteristic between included and exclude cases to evaluate the randomness of excluded. Second, the small proportion of diabetic nephropathy as an etiology of ESKD in our cohort might limit the finding’s generalization. Third, although the study is a relatively large cohort on association of PDELC and mortality in a recent 10 years, the number of patients who died was small, which limited our ability to describe and analyze the mortality of patients with PDAP in this study.

## Conclusion

Among patients with PDAP, an increased PDELC on day 5 (≥2000 × 10^6^/L) was associated with a significantly greater risk of 60-day and half-year mortality. An increased PDELC on day 5 (≥100 × 10^6^/L) was associated with a greater risk of treatment failure. Increased PDELC-5D also contributed to the lengthening of in-hospital LOS.

## Supplementary Material

Supplemental Material
