# Supplementary material for: Association between dialysis effluent leukocyte count after initial antibiotic treatment and outcomes of patients with peritoneal dialysis-associated peritonitis: a retrospective study
Source: Ren Fail. 2023 Sep 22;45(2):2258990. doi: 10.1080/0886022X.2023.2258990 (PMC10519253; doi:10.1080/0886022X.2023.2258990)
Supplement: Supplemental Material [file IRNF_A_2258990_SM9349.pdf]

Table S1. Baseline Characteristics of excluded (N=48) and included (N=549) cases.

Variables	All (n=597)	Excluded (n=48)	Included (n=549)
**sex,** n (%)			
female	249(41.8)	18 (38.3)	231 (42.2)
male	346 (58.2)	29 (61.7)	317 (57.8)
**Age (years)**	53.2 ± 14.5	49.9 ± 19.4	53.4 ± 14.0
**Etiology of ESRD**, n (%)			
Chronic glomerulonephritis	431 (72.3)	36 (76.6)	395 (71.9)
Diabetes nephropathy	21 (3.5)	2 (4.3)	19 (3.5)
Hypertensive nephropathy	56 (9.4)	5 (10.6)	51 (9.3)
Obstructive nephropathy	51 (8.6)	3 (6.4)	48 (8.7)
Lupus nephritis	13 (2.2)	0 (0)	13 (2.4)
Gouty nephropathy	13 (2.2)	0 (0)	13 (2.4)
Chronic interstitial nephritis	6 (1.0)	1 (2.1)	5 (0.9)
Polycystic kidney disease	5 (0.8)	0 (0)	5 (0.9)
**Comorbidities**, n (%)			
Clinical atherosclerotic vascular disease	125 (21.0)	7 (14.9)	118 (21.5)
Diabetes	35 (5.9)	2 (4.3)	33 (6.0)
**Comorbidity Infections,** n (%)			
respiratory tract infection	125 (21.0)	11(23.4)	114 (20.8)
Gastroenteritis	14 (2.3)	3 (6.4)	11 (2.0)
sepsis	5 (0.8)	0 (0)	5 (0.9)
cholecystitis	6 (1.0)	0 (0)	6 (1.1)
Tunnel infection	4 (0.7)	0 (0)	4 (0.7)
others	6 (1.0)	0 (0)	6 (1.1)
**PD duration (months)**, Mean ± SD	35.6 ± 31.3	38.6 ± 33.2	35.3 ± 31.1
**APD,** n (%)	27 (4.5)	0 (0)	27 (4.9)
**Laboratory variables**			
HB (g/L), Mean ± SD	103.1±21.6	102.4 ± 24.7	103.2 ± 21.4
WBC ($\times10^9$/L), Mean ± SD	10.0± 5.0	9.8± 6.0	10.0 ± 4.9
Albumin (g/L), Mean ±SD	28.9± 5.3	27.5 ± 5.4	29.0 ± 5.3
Serum Potassium (mmol/L), Mean ± SD	3.5 ± 0.7	3.4 ± 0.7	3.5 ± 0.7
Serum phosphorus (mmol/L), Mean ± SD	1.5 ± 0.5	1.6 ± 0.5	1.5 ± 0.5
CRP, (mg/L) Median (IQR)	86.4 (38.2, 136.0)	80.1 (27.9, 125.2)	88.3 (38.8, 136.5)
PCT, (ng/ml) Median (IQR)	5.0 (1.1, 26.5)	5.6 (1.8, 41.5)	4.9 (1.1, 25.2)
PDELC on day 1 ($\times10^6$/L）, Median (IQR)	1856.0 (600.0, 4200.0)	3500.0 (805.0, 6320.0)	1800.0 (590.0, 4100.0)
**Organisms**			
**Culture negative**, n (%)	144 (24.1)	12 (25.0)	132 (24.0)
**Gram positive**, n (%)	263 (44.1)	23 (47.9)	240 (43.7)

Information Classification: General

Coagulase-negative staphylococcus	92 (15.4）	9 (18.8)	83 (15.1)
Staphylococcus aureus	19 (3.2）	1 (2.1)	18 (3.3)
MRSA	5 (0.8)	1 (2.1)	4 (0.7)
Eenterococcus	17 (2.8)	3 (6.2)	14 (2.6)
**Gram negative**, n (%)	176 (29.5)	10 (20.8)	166 (30.2)
Escherichia coli	96 (16.1)	6 (12.5)	90 (16.4)
Pseudomonas	26 (4.4)	1(2.1)	25 (4.6)
Klebsiella	32 (5.4)	3 (6.2)	29 (5.3)
**Fungus**, n (%)	35 (5.9)	2 (4.2)	33 (6.0)
**Multi-organisms**, n (%)	28 (4.7)	1 (2.1)	27 (4.9)
**Clinical outcomes**			
Catheter removal, n (%)	61 (10.2)	6 (12.8)	55 (10.0)
treatment failure, n (%)	89 (14.9)	21 (5.7)	78 (14.2)
60-day mortality, n (%)	39 (6.5)	6 (12.8)	33 (6.0)
Half-year mortality, n (%)	59 (9.9)	7 (14.9)	52 (9.8)
in hospital LOS (day), Median (IQR)	10.0 (7.0, 15.0)	11.0 (7.0, 17.5)	10.0(7.0, 15.0)

Abbreviations: PD, peritoneal dialysis; PDELC, peritoneal dialysis effluent leukocyte count; HB, Hemoglobin; WBC, White blood cell count; CRP, C-reactive protein; PCT, Procalcitonin; LOS: Length of stay.

In this study, 48 cases were excluded due to missing data on the PDELC on day 5. In order to evaluate the randomness of missing data, we compared the baseline characteristics of included and excluded cases There is no obvious difference in the baseline characteristics between the two groups, except for mortality. Among the excluded cases, a total of six patients died within 60 days, in which three patients gave up the treatment and died after leaving hospital, and one patient with septic shock and heart failure died on day 4.

Table S2. The distributions of variables with missing data and the multiple imputation data.

variable	Number (%) with missing data	Complete case	Multiple imputation
CRP	41 (6.88%)	88.3 [38.7, 136.5]	89.9 [38.98, 138.0]
PCT	38 (6.38%)	4.9 [1.1, 25.2]	5.14 [1.13, 25.34]
Serum albumin	4 (0.67%)	29.0 (5.30)	29.01 (5.30)
serum phosphorus	4 (0.67%)	1.50 (0.50)	1.54 (0.52)

Notes: data presented are mean ± SD, median (Q1–Q3), or N (%).

The distributions of all the variables of missing data were of similar values with the imputation data (Table S2).

Information Classification: General

Table S3. Multivariable logistic regression models evaluating the association between PDELC on day 5 and clinical outcomes (using a complete-case data).

Variable	n. total	n. event (%)	Non-adjusted Model		Model 1		Model 2		Model 3	
			OR (95%CI)	P-value	OR (95%CI)	P-value	OR (95%CI)	P-value	OR (95%CI)	P-value
**60-day mortality**										
PDELC on day 5 (continuous), per 100×10⁶/L	549	33 (6)	1.05 (1.03,1.07)	<0.001	1.04 (1.03, 1.06)	<0.001	1.04 (1.02,1.06)	<0.001	1.04 (1.02-1.07)	<0.001
<100×10⁶/L	370	10 (2.7)	Ref		Ref		Ref		Ref	
100- 2000×10⁶/L	134	9 (6.7)	2.59 (1.03, 6.53)	0.043	2.41 (0.95, 6.13)	0.064	1.37 (0.46, 4.02)	0.57	1.36 (0.46, 4.03)	0.578
⩾2000×10⁶/L	45	14 (31.1)	16.26 (6.67, 39.61)	<0.001	14.05 (5.68, 34.74)	<0.001	8.03 (2.80, 23.06)	<0.001	9.48 (3.04, 29.50)	<0.001
**Half-year mortality**										
PDELC on day 5 (continuous), per 100×10⁶/L	549	52(9.5)	1.04 (1.03, 1.06)	<0.001	1.04 (1.02, 1.06)	<0.001	1.04 (1.02, 1.06)	<0.001	1.04 (1.02, 1.07)	<0.001
<100×10⁶/L	370	20 (5.4)	Ref		Ref		Ref		Ref	
100- 2000×10⁶/L	134	16 (11.9)	2.37 (1.19, 4.73)	0.014	2.26 (1.13, 4.54)	0.022	1.11 (0.48, 2.55)	0.812	1.10 (0.46, 2.59)	0.835
⩾2000×10⁶/L	45	16 (35.6)	9.66 (4.52, 20.62)	<0.001	8.58 (3.97, 18.56)	<0.001	4.87 (2.00, 11.87)	<0.001	6.85 (2.52, 18.66)	<0.001
**Treatment failure**										
PDELC on day 5 (continuous), per 100×10⁶/L	549	78 (14.2)	1.05 (1.03, 1.07)	<0.001	1.05 (1.03, 1.07)	<0.001	1.04 (1.02, 1.06)	<0.001	1.04 (1.02, 1.07)	<0.001
<100×10⁶/L	370	21 (5.7)	Ref		Ref		Ref		Ref	
100-2000×10⁶/L	134	36 (26.9)	6.1 (3.41, 10.94)	<0.001	5.92 (3.28, 10.67)	< 0.001	2.81 (1.27, 6.20)	0.011	2.41 (1.05, 5.52)	0.037
⩾2000×10⁶/L	45	21 (46.7)	14.54 (6.99, 30.26)	<0.001	13.05(6.21, 27.43)	<0.001	6.71 (2.57, 17.51)	<0.001	7.13 (2.49, 20.45)	<0.001

NOTES: 1) using a complete-case data)

2) Adjust I model adjusts for age and PD-duration; Adjust II model adjusts for adjusts I + Fungal peritonitis+ diabetes mellitus+ CRP; Adjust III model adjusts for adjusts II + albumin + Gram-negative infection + multi-organism infection.

Table S4. Multivariable linear regression models evaluating the association between PDELC on day 5 and in hospital LOS (using a complete-case data).

Variable	n. total	Non-adjusted Model		Model 1		Model 2		Model 3	
		β (95%CI)	P-value	β (95%CI)	P-value	β (95%CI)	P-value	β (95%CI)	P-value
PDELC-5D (continuous), per 100 ×10⁶/L	549	0.30 (0.25,0.35)	<0.001	0.29 (0.24,0.34)	<0.001	0.23 (0.18, 0.28)	<0.001	0.23 (0.18, 0.28)	<0.001
<100×10⁶/L	356	Ref		Ref		Ref		Ref	
100 - 2000 ×10⁶/L	108	6.11 (4.58, 7.64)	<0.001	6.11 (4.58, 7.64)	< 0.001	3.68 (2.18, 5.17)	< 0.001	3.54 (2.06, 5.03)	< 0.001
⩾2000 ×10⁶/L	27	13.42 (11.02, 15.82)	<0.001	13.16 (10.75, 15.56)	< 0.001	9.81 (7.44, 12.18)	< 0.001	9.87 (7.48, 12.37)	< 0.001

NOTES: 1) using a complete-case data)

2) Adjust I model adjusts for age and PD-duration; Adjust II model adjusts for adjusts I + Fungal peritonitis+ diabetes mellitus+ CRP; Adjust III model adjusts for adjusts II + albumin + Gram-negative infection + multi-organism infection.

Information Classification: General

Table S5. Baseline of characteristics and outcomes of early-onset and later-onset PDAP cases.

Variables	All (n=549)	Early-onset (<12 months) (n=156)	Late-onset (≥12 months) (n=393)
**Sex**			
Female, n (%)	231(42.2)	67 (42.9)	164 (41.8)
Male, n (%)	317 (57.8)	89 (57.1)	228 (58.2)
**Age** (years)	53.4 ± 14.0	53.9 ±16.1	53.3 ±13.1
**APD**, n (%)	27 (4.9)	8 (5.1)	19 (4.8)
**Laboratory variables**			
Alb (g/L), Mean±DS	29.0±5.3	29.5±5.5	28.8±5.2
CRP (mg/L), Median (IQR)	88.3 (38.8, 136.5)	82.6 (23.4, 122.8)	91.1 (43.4, 142.0)
PCT (pg/ml), Median (IQR)	4.9 (1.1, 25.2)	5.0 (1.0, 24.9)	4.9 (1.1, 25.3)
PDELC on day 1(×106/L), Median (IQR)	1800.0 (590.0, 4100.0)	1800.0 (612.5, 3620.0)	1800.0 (587.0, 4160.0)
PDELC on day 5(×106/L), Median (IQR)	30.0 (8.0, 220.0)	25.0 (8.0, 92.5)	40.0 (10.0, 330.0)
**Organisms**			
**Culture negative**, n (%)	132 (24.0)	44 (28.2)	88 (22.4)
**Gram_ positive**, n (%)	240 (43.7)	68 (43.6)	172 (43.8)
Coagulase-negative staphylococcus	83 (15.1)	20 (12.8)	63 (16)
Staphylococcus aureus	18 (3.3)	5 (3.2)	13 (3.3)
MRSA	4 (0.7)	2 (1.3)	2 (0.5)
Eenterococcus	14 (2.6)	2 (1.3)	12 (3.1)
**Gram negative**, n (%)	166 (30.2)	42 (26.9)	124 (31.6)
Escherichia coli	90 (16.4)	18 (11.5)	72 (18.3)
Pseudomonas	25 (4.6)	7 (4.5)	18 (4.6)
Klebsiella	29 (5.3)	9 (5.8)	20 (5.1)
**Fungus**, n (%)	33 (6.0)	5 (3.2)	28 (7.1)
**Multi-organisms**, n (%)	27 (4.9)	4 (2.6)	23 (5.9)
**Clinical outcomes**			
treatment failure, n (%)	78 (14.2)	12 (7.7)	66 (16.8)
60-day mortality, n (%)	33 (6.0)	3 (1.9)	30 (7.6)
Half-year mortality, n (%)	52 (9.5)	6 (3.8)	46 (11.7)
in hospital LOS (day), Median (IQR)	10.0 (7.0, 15.0)	9.0 (7.0, 14.0)	10.0(7.0, 15.0)

Abbreviations: PD, peritoneal dialysis; PDELC, peritoneal dialysis effluent leukocyte count; HB, Hemoglobin; WBC, White blood cell count; CRP, C-reactive protein; PCT, Procalcitonin; LOS: Length of stay. APD, automated peritoneal dialysis;

Information Classification: General

Table S6. The multivariable linear regression models evaluating the association between PDELC on day 5 and Logarithmic transformed LOS in hospital.

Variable	n. total	Non-adjusted Model		Model 1		Model 2		Model 3	
		β (95%CI)	P-value	β (95%CI)	P-value	β (95%CI)	P-value	β (95%CI)	P-value
PDELC on day 5 (continuous), per 100 ×10⁶/L	549	0.02 (0.02,0.02)	<0.001	0.02 (0.01,0.02)	<0.001	0.01 (0.01, 0.02)	<0.001	0.01(0.01, 0.02)	<0.001
<100×10⁶/L	370	Ref		Ref		Ref		Ref	
100 - 2000 ×10⁶/L	134	0.5 (0.4, 0.6)	<0.001	0.51 (0.4, 0.61)	< 0.001	0.4 (0.3, 0.51)	< 0.001	0.39 (0.28, 0.49)	< 0.001
⩾2000 ×10⁶/L	45	0.87 (0.71, 1.03)	<0.001	0.86 (0.69, 1.02)	< 0.001	0.67 (0.50, 0.83)	< 0.001	0.64 (0.47, 0.81)	< 0.001

Note: 1) dependent variable = ln(LOS).

2) Adjust I model adjusts for age and PD-duration; Adjust II model adjusts for adjusts I + Fungal peritonitis+ diabetes mellitus+ CRP; Adjust III model adjusts for adjusts II + albumin + Gram-negative infection + multi-organism infection.

Table S7. Excluding episodes with culture-negative result, multivariable logistic regression models evaluating the association between PDELC on day 5 and clinical outcomes.

Variable	n. total	n. event (%)	Non-adjusted Model		Model 1		Model 2		Model 3	
			OR (95%CI)	P-value	OR (95%CI)	P-value	OR (95%CI)	P-value	OR (95%CI)	P-value
**60-day mortality**										
PDELC on day 5 (continuous), per 100×10⁶/L	417	30(7.2)	1.05(1.03,1.06)	<0.001	1.04(1.03, 1.06)	<0.001	1.03(1.01,1.05)	0.001	1.03 (1.01-1.05)	0.001
<100×10⁶/L	277	9 (3.2)	Ref		Ref		Ref		Ref	
100- 2000×10⁶/L	98	7 (7.1)	2.29 (0.83, 6.33)	0.11	2.14 (0.77, 5.95)	0.146	1.76 (0.6, 5.15)	0.305	1.71 (0.58, 5.02)	0.328
⩾2000×10⁶/L	42	14 (33.3)	14.89(5.91, 7.49)	<0.001	12.97 (5.06, 33.21)	<0.001	10.37(3.86, 27.87)	<0.001	10.15(3.75, 27.52)	<0.001
**Half-year mortality**										
PDELC on day 5 (continuous), per 100×10⁶/L	417	45(10.8)	1.04 (1.03, 1.06)	<0.001	1.04 (1.02, 1.06)	<0.001	1.03 (1.01, 1.05)	0.002	1.03 (1.01, 1.05)	0.002
<100×10⁶/L	277	18 (6.5)	Ref		Ref		Ref		Ref	
100- 2000×10⁶/L	98	11 (11.2)	1.82 (0.83, 4.0)	0.137	1.71 (0.77, 3.79)	0.187	1.38 (0.59, 3.19)	0.459	1.29 (0.55, 3.02)	0.486
⩾2000×10⁶/L	42	16 (38.1)	8.85 (4.04, 19.41)	<0.001	7.78 (3.49, 17.32)	<0.001	6.1 (2.63, 14.14)	0.001	6.02(2.52, 14.36)	0.001
**Treatment failure**										
PDELC on day 5 (continuous), per 100×10⁶/L	417	68 (16.3)	1.05 (1.03, 1.07)	<0.001	1.05 (1.03, 1.06)	<0.001	1.03 (1.01, 1.05)	0.002	1.03 (1.01, 1.05)	0.011
<100×10⁶/L	277	17 (6.1)	Ref		Ref		Ref		Ref	
100-2000×10⁶/L	98	31 (31.6)	7.08 (3.70, 13.55)	<0.001	6.87 (3.57, 13.23)	< 0.001	6.13 (3.12, 12.05)	<0.001	5.98 (3.03, 11.8)	<0.001
⩾2000×10⁶/L	42	20 (47.6)	13.9 (6.38, 30.31)	<0.001	12.63(5.72, 27.88)	<0.001	10.66(4.70, 24.20)	<0.001	10.58(4.62, 24.24)	<0.001

Note: 1) Episodes with culture-negative result were excluded.

2) Adjust I model adjusts for age and PD-duration; Adjust II model adjusts for adjusts I + Fungal peritonitis+ diabetes mellitus+ CRP; Adjust III model adjusts for adjusts II + albumin + Gram-negative infection + multi-organism infection.

Information Classification: General

Table S8. Excluding episodes with MRSA, Pseudomonas spp., and fungal peritonitis, multivariable logistic regression models evaluating the association between PDELC on day 5 and clinical outcomes.

Variable	n. total	n. event (%)	Non-adjusted Model		Model 1		Model 2		Model 3	
			OR (95%CI)	P-value	OR (95%CI)	P-value	OR (95%CI)	P-value	OR (95%CI)	P-value
**60-day mortality**										
PDELC on day 5 (continuous), per 100×10^6/L	491	19(3.9)	1.04(1.01,1.06)	0.001	1.03(1.01, 1.06)	0.004	1.03(1.01,1.06)	0.01	1.03 (1.00-1.05)	0.036
<100×10^6/L	356	9 (2.5)	Ref		Ref		Ref		Ref	
100- 2000×10^6/L	108	6 (5.6)	2.27 (0.79, 6.52)	0.129	2.16 (0.74, 6.31)	0.161	1.71 (0.54, 5.47)	0.363	1.51 (0.45, 5.00)	0.502
⩾2000×10^6/L	27	4(14.8)	6.71(1.92, 23.43)	0.003	6.06 (1.71, 21.43)	0.005	5.06(1.35, 18.89)	0.016	4.38(1.05, 18.25)	0.042
**Half-year mortality**										
PDELC on day 5 (continuous), per 100×10^6/L	491	35 (7.1)	1.04 (1.02, 1.06)	<0.001	1.03 (1.01, 1.05)	<0.001	1.03 (1.01, 1.05)	0.004	1.03 (1.01, 1.05)	0.009
<100×10^6/L	356	18 (5.1)	Ref		Ref		Ref		Ref	
100- 2000×10^6/L	108	11 (10.2)	2.13 (0.97, 4.66)	0.059	2.13 (0.96, 4.74)	0.064	1.78 (0.76, 4.18)	0.186	1.53 (0.63, 3.70)	0.35
⩾2000×10^6/L	27	6 (22.2)	5.37 (1.93, 14.93)	0.001	5.03 (1.79, 14.18)	0.002	4.32 (1.48, 12.57)	0.007	4.75(1.44, 15.67)	0.011
**Treatment failure**										
PDELC on day 5 (continuous), per 100×10^6/L	491	40 (8.1)	1.05 (1.03, 1.07)	<0.001	1.05 (1.02, 1.07)	<0.001	1.04 (1.02, 1.06)	<0.001	1.04 (1.02, 1.06)	0.001
<100×10^6/L	356	14 (3.9)	Ref		Ref		Ref		Ref	
100-2000×10^6/L	108	18 (16.7)	4.89 (2.34, 10.2)	<0.001	4.56 (2.15, 9.64)	< 0.001	3.93 (1.78, 8.69)	0.001	3.55 (1.57, 8.02)	0.002
⩾2000×10^6/L	27	8 (29.6)	10.29(3.85,27.51	<0.001	9.15 (3.38, 24.74)	<0.001	8.02(2.88, 22.34)	<0.001	7.89(2.56, 24.27)	<0.001

Note: 1) Episodes with MRSA, Pseudomonas spp., and fungal peritonitis were excluded.

2) Adjust I model adjusts for age and PD-duration; Adjust II model adjusts for adjusts I + Fungal peritonitis+ diabetes mellitus+ CRP; Adjust III model adjusts for adjusts II + albumin + Gram-negative infection + multi-organism infection.

Information Classification: General

Table S9. E-values to assess unmeasured confounding in the Primary and Secondary outcomes.

	statistic	
outcome	E-value	E-value Lower Limit
60-day mortality	13.46	4.09
Half-year mortality	9.412	3.27
Treatment failure	11.02	3.558

Table S10. 60-day mortality multivariate logistic regression.

Variable	Odds Ratios	95% confidence interval	P Value
Age	1.01	0.98, 1.05	0.481
PD duration	1.01	1, 1.02	0.185
CRP	1	1,1.01	0.176
Alb	0.98	0.91, 1.05	0.553
Gram-negative	0.87	0.33, 2.28	0.774
Multi-organisms	1.47	0.38, 5.72	0.576
Fungal peritonitis	5.98	1.86, 19.23	0.003

PDELC on day 5 ⩾ 2000 $\times 10^6$/L was associated with an increase in 60-day mortality by multivariable analysis in the full adjusted model (OR, 6.99 [95% CI 2.33-20.92]). The E-value was OR >13.46, meaning that residual confounding could explain the observed association if there exists an unmeasured covariate having a OR⩾13.46 with both 60-day mortality and PDELC on day 5. Significant known and measured risk factors for 60-day mortality within the multivariable logistic regression model included age (OR, 1.01 [95% CI, 0.98-1.05]), PD duration (OR, 1.01 [95% CI, 1-1.02]), diabetes mellitus (OR, 1.48 [95% CI, 0.38-5.71]), serum albumin (OR, 0.98 [95% CI, 0.91-1.05])，CRP (OR, 1.0 [95% CI, 1-1.01]), fungal peritonitis (OR, 5.98 [95% CI, 1.86-19.23]), multi-organisms peritonitis (OR, 1.47 [95% CI, 0.38-5.72]), Gram-negative organisms (OR, 0.87 [95% CI, 0.33-2.28). Therefore, it is unlikely that an unmeasured or unknown confounder would have a substantially greater effect on 60-day mortality (OR exceeding 13.46) than these known risk factors.

Information Classification: General

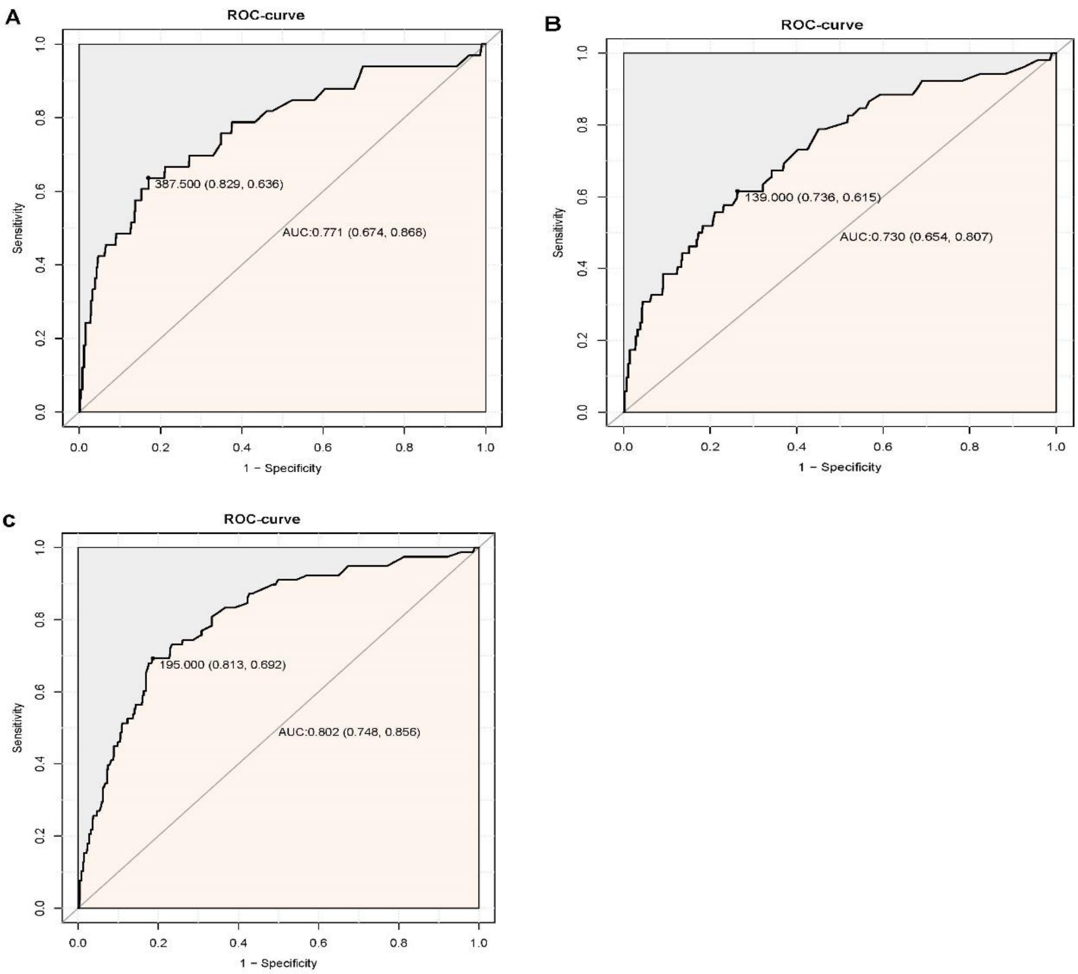

Figure S1: Receiver operating characteristic (ROC) curve of clinical outcomes and PDELC on day 5, constructed to evaluate the sensitivity and 1- specificity of PDELC on day 5 in the predict 60-day mortality (A), half-year mortality (B), and treatment failure (C).

Information Classification: General

Table S11. Multivariable logistic regression models evaluating the association between the declining ratio of PDELC on day 5 and clinical outcomes.

Variable	n. total	n. event (%)	Non-adjusted Model		Full adjusted Model	
			OR (95%CI)	P-value	OR (95%CI)	P-value
**60-day mortality**						
Decline ratio of PDELC (continuous), per 0.1	549	33(6)	0.99(0.99,1.0)	0.066	1.0(0.99, 1.0)	0.419
>0.9	377	12 (3.2)	Ref		Ref	
0.6-0.9	76	4 (5.3)	1.69 (0.53, 5.39)	0.375	1.08 (0.31, 3.83)	0.903
0-0.6	49	6 (12.2)	4.24 (1.52, 11.88)	0.006	2.74 (0.91, 8.21)	0.072
< 0	47	11 (23.4)	9.29 (3.83, 22.56)	<0.001	2.79 (0.93, 8.4)	0.067
**Half-year mortality**						
Decline ratio of PDELC (continuous), per 0.1	549	52 (9.5)	1.01 (0.99, 1.04)	0.229	1.01 (0.98, 1.03)	0.579
>0.9	377	19 (5)	Ref		Ref	
0.6-0.9	76	9 (11.8)	2.53(1.1, 5.83)	0.029	1.83 (0.75, 4.46)	0.187
0-0.6	49	10 (20.4)	4.83 (2.1, 11.12)	<0.001	3.65 (1.5, 8.9)	0.004
< 0	47	14 (29.8)	7.99 (3.68, 17.39)	<0.001	3.3 (1.3, 8.41)	0.012
**Treatment failure**						
Decline ratio of PDELC (continuous), per 0.1	549	78 (14.2)	1.01 (0.99, 1.03)	0.19	1.0 (0.97, 1.03)	0.94
>0.9	377	24(6.4)	Ref		Ref	
0.6-0.9	76	14 (18.4)	3.32 (1.63, 6.77)	0.001	2.36 (0.98, 5.66)	0.055
0-0.6	49	14 (28.6)	5.88 (2.79, 12.39)	<0.001	4.27 (1.7, 10.69)	0.002
< 0	47	26 (55.3)	18.21 (8.97, 36.98)	<0.001	5.12 (1.85, 14.23)	0.002

Note: 1) decline ratio of PDELC = (PDELC on day 1− PDELC on day 5)/PDELC on day 1

2) Full adjusted model adjusts for age+ PD-duration+ Fungal peritonitis+ diabetes mellitus+ CRP + albumin + Gram-negative bacteria peritonitis + multi-organism peritonitis.

Information Classification: General

Table S12. Clinical characteristic and outcomes of patients removed catheter.

**Variables**	**All cases removed catheter (n=55)**	**PDELC on day 5 <2000 (n=40)**	**PDELC on day 5 ≥2000 (n=15)**
Female, n (%)	22 (40.0)	17 (42.5)	5 (33.3)
Age, Mean±SD	55.6±11.8	56.7±11.3	52.5±13.0
CRP (mg/L), Median (IQR)	119.9 (80.2, 175.2)	112.3 (77.7, 179.8)	143.0 (92.8,162.5)
Fungal PDAP, n(%)	30 (54.5)	21 (52.5)	9 (60)
Time of removal (days), Median (IQR)	14.0 (8.0, 19.5)	14.0 (9.5, 19.0)	15.0 (8.0, 21.0)
60-day mortality, n(%)	11 (20.0)	3 (7.5)	8 (53.5)
Half-year mortality, n(%)	15 (27.3)	6 (15)	9 (60)

Other information about patients who were removed catheter: Among the 33 patients who died within 60 days, 11 cases were removed the catheter, the timing of removal is 15 [11, 19] (Median [IQR]) days.

Information Classification: General
